# Poly(3,4-ethylenedioxythiophene) Nanorod Arrays-Based Organic Electrochemical Transistor for SARS-CoV-2 Spike Protein Detection in Artificial Saliva

**DOI:** 10.1021/acssensors.4c03207

**Published:** 2025-03-13

**Authors:** Syed Atif Ali, Ying-Lin Chen, Hsueh-Sheng Tseng, Hailemichael Ayalew, Jia-Wei She, Bhaskarchand Gautam, Hsiung-Lin Tu, Yu-Sheng Hsiao, Hsiao-hua Yu

**Affiliations:** a Department of Materials Science and Engineering, National Taiwan University of Science and Technology, Taipei 106335, Taiwan; b Smart Organic Materials Laboratory, Institute of Chemistry, Academia Sinica, Nankang, Taipei 11529, Taiwan; c Institute of Chemistry, Academia Sinica, Nankang, Taipei 11529, Taiwan; d Sustainable Chemical Science & Technology, Taiwan International Graduate Program (TIGP), Academia Sinica, Nankang, Taipei 11529, Taiwan; e Department of Applied Chemistry, National Yang Ming Chiao Tung University, Hsinchu 30010, Taiwan; f Department of Engineering and System Science, National Tsing Hua University, Hsinchu 30010, Taiwan

**Keywords:** coronavirus disease 2019 (COVID-19), severe acute respiratory syndrome coronavirus 2 (SARS-CoV-2), organic electrochemical transistors (OECTs), poly(3,4-ethylenedioxythiophene) (PEDOT), biosensor

## Abstract

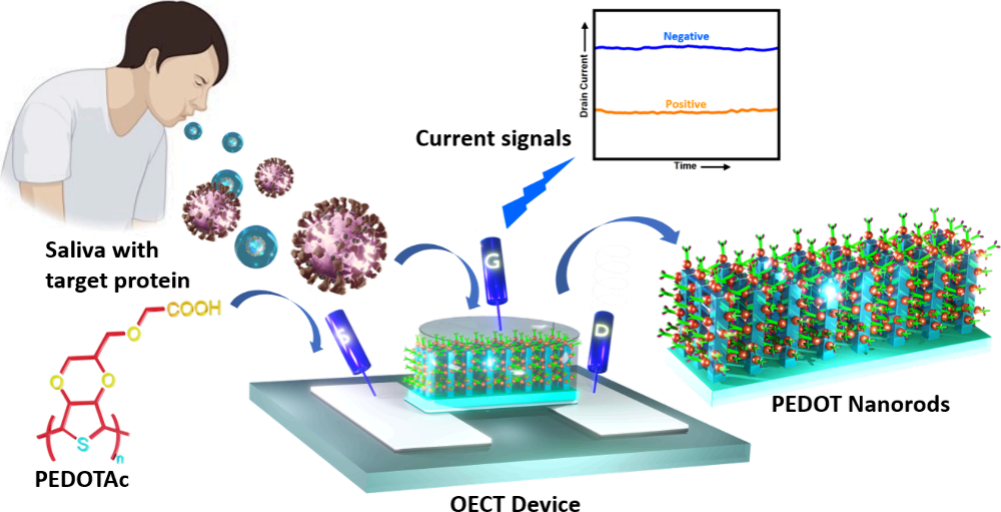

The outbreak and continued spread of coronavirus disease 2019 (COVID-19) have significantly threatened public health. Antibody testing is essential for infection diagnosis, seroepidemiological analysis, and vaccine evaluation. However, achieving convenient, fast, and accurate detection remains challenging in this prolonged battle. This study reports a highly sensitive severe acute respiratory syndrome coronavirus 2 (SARS-CoV-2) spike protein detection platform based on organic electrochemical transistors (OECTs) for biosensing applications. We developed a nanostructured poly(3,4-ethylenedioxythiophene) (PEDOT) conductive polymer with the carboxylic acid functional group (PEDOTAc) for modifying specific antibodies on an OECT channel for the detection of the COVID-19 spike protein. The OECT device features a channel composed of a PEDOT:polystyrenesulfonate (PEDOT:PSS) bottom layer, with the upper layer decorated with PEDOTAc nanorod arrays via the oxidative polymerization and a trans-printing method. Our novel PEDOTAc nanorod array-based OECT device exhibits promising potential for future healthcare and point-of-care sensing due to its rapid response, high sensitivity, and high accuracy. Through optimization, we achieved specific detection of the SARS-CoV-2 spike protein within minutes, with a detectable region from 10 fM to 100 nM. These biosensors hold significant promise for use in the diagnosis and prognosis of COVID-19.

Since January 2020, the global spread of coronavirus disease (COVID-19), caused by severe acute respiratory syndrome coronavirus 2 (SARS-CoV-2), has marked the emergence of a highly contagious infection. The initial case was identified in East Asia in December 2019, leading to its subsequent worldwide spread and the declaration of a pandemic.^1^ Despite significant efforts over the past few years, the virus continues to spread severely in numerous countries. COVID-19 not only presents a substantial threat to public health but also imposes extensive economic and social burdens globally.^2^ Given its global impact, COVID-19 stands as an important issue. The absolute magnitude of the infected cases during the pandemic necessitates an immediate response to control its spread. Early detection and treatment are pivotal in containing the outbreak. Identifying COVID-19 can be based on symptoms and subsequently confirmed through real-time reverse transcription–polymerase chain reaction (RT-PCR) or other nucleic acid testing of contaminated secretions. Serological tests, which detect antibodies produced by the body in response to infection, offer a means to diagnose previous infections.^3,4^

The swift and precise diagnosis of novel coronavirus infection is crucial for selecting suitable treatment methods to save human lives and curb virus transmission. Currently, most coronavirus diagnoses primarily involve nucleic acid detection and antigen–antibody detection. The spike protein of COVID-19 testing holds significance in various aspects such as a prime target for diagnostic assays, and it may help in updating the antigenic targets in diagnostic tests.^5,6^ Additionally, it plays a vital role in seroepidemiological studies, enabling the analysis of infection rates and population immunity rates in various geographical areas.^7^ The initial step in testing COVID-19 involves the accurate detection of SARS-CoV-2 facilitated by RT-PC. RT-PCR identifies SARS-CoV-2 nucleic acids found in nasopharyngeal fluids.

In line with this, different biosensors have been developed for detecting influenza, human immunodeficiency virus, and other viral diseases. Initially limited by low sensitivity and specificity, these shortcomings were overcome by plasmonic nanoparticles (NPs, e.g., gold and silver), metal oxide NPs, and field effect transistor (FET) sensors.^8−11^ Nevertheless, human saliva has gained attention as an alternative diagnostic medium for detecting infections due to the presence of SARS-CoV-2 in saliva. The other samples for COVID-19 testing include faces and radiological examinations.^12,13^ SARS-CoV-2 can independently colonize the oral cavity and salivary glands via several pathways^14,15^ with transmission through saliva droplets perpetuated by activities such as speaking and sneezing.^16^ PCR testing of saliva has been shown to have comparable^17,18^ or higher sensitivity and stability^19^ than PCR testing for COVID-19 using nasal or nasopharyngeal swabs.

Currently, the clinical diagnosis of COVID-19 relies on a combination of chest CT and RT-PCR results. Outside a clinical setting, RT-PCR testing comprises the vast majority of surveillance testing in the workplace or within schools. However, nucleic acid amplification tests may be problematic with poorly timed specimen collection, low-quality samples, a requirement for trained laboratory technicians, and long wait times to generate the results. The gold standard RT-qPCR (quantitative PCR) is time-consuming (4–6 h), not including the time to transport the specimens to the laboratory, which can take days.^20^

Many researchers recently developed various surfaces based on electrochemical sensors such as cyclic voltammetry (CV),^21^ electrochemical impedance spectroscopy (EIS),^22^ square wave voltammetry (SWV),^23^ and chronoamperometry.^24^ These techniques are highly effective, but they often require complex setups or sophisticated data interpretation.

In recent years, organic electrochemical transistors (OECTs) have been recognized as high-performance transducers and amplifiers that can convert biological signals into electrical signals^25^ to detect various biomolecules, including microbes,^26,27^ nucleic acids,^28−30^ proteins,^31,32^ hormones,^33,34^ and metabolite.^35,36^ Particularly, OECTs based on poly(3,4-ethylenedioxythiophene):polystyrenesulfonate (PEDOT:PSS) became versatile due to their signal amplification properties, ease of miniaturization, and exceptional biocompatibility when used as bioelectronic interfaces.^37−40^

Many OECTs have been used as signal transducing elements for signal amplification within the sensors, including a carbon cloth gate electrode OECT for the detection of ascorbic acid and dopamine,^41,42^ a paper-based flexible OECT for the detection of glucose and H_2_O_2_,^43^ a textile-based wearable sweat OECT sensor for sensing K^+^ and Ca^2+^ ions, a self-powered ion-sensing OECT for wearable, portable, and self-powered sensors, an OECT wearable patch for Ca^2+^- and ammonium-ion sensing in human perspiration, and a microfluidic integrated OECT for ultrasensitive detection of a metabolite.^44,45^ The OECTs were first constructed by a device with a microelectrode array that can work as a transistor to amplify the tiny current when immersed in an electrolyte solution.^46^

Various substrates used for developing biosensors present challenges, including complex electrode fabrication, a limit of detection (LOD) that depends on antibody immobilization, aggregation issues, and mass production constraints.^47^ Numerous studies have shown that material shapes and nanostructures can significantly enhance sensing properties compared to conventional planar thin-film or nanocomposite-based sensors.^48−51^ For instance, Pt/graphene,^52,53^ functionalized carbon nanotubes,^54−57^ silver nanoflowers,^58^ and silicon nanowire electrode arrays^59,60^ have demonstrated excellent sensitivity in electrochemical tests.

Here, we report a novel OECT-based biosensor for the detection of the SARS-CoV-2 spike protein. ACE2 is immobilized on the channel through the biotin–streptavidin strategy, and the SARS-CoV-2 spike protein is bound with the receptor through receptor-antigen reaction during incubation, leading to a response of the device to detect signals. Our device can detect the SARS-CoV-2 spike protein with an ultralow detection limit of 0.1 fM in PBS solution and 10 pM in a saliva sample. The detection range in saliva is from 10 pM to 100 nM with a good linear relationship. The ultralow detection limit in saliva proves the analytical sensitivity of the device.

## Experimental Section

### Chemicals and Material Characterizations

All experiments utilized analytical-grade reagents. The synthesis of carboxyl-functionalized EDOT (EDOTAc) followed our laboratory’s established protocol.^61^*para*-Toluenesulfonate hexahydrate [Fe(III)TOS] was procured from Sigma-Aldrich, and imidazole (IM) was obtained from TCI. PEDOT:PSS (PH1000) aqueous solution was sourced from Uniregion Biotech. Methanol (MeOH) and DMSO were acquired as HPLC grade from JT Baker. Streptavidin (SA, 1 mg/mL) was obtained from Sigma. Biotinylated human ACE2/Angiotensin-Converting enzyme 2 Protein (His-Tag) and SARS-CoV-2 (2019-nCoV) Spike RBD recombinant protein were purchased from Sino Biological (Beijing, China). The 10× PBS consists of 137 mM NaCl, 2.7 mM KCl, and 10 mM phosphate buffer with a pH of 7.4. This PBS buffer (pH 7.4) was used for the dissolution of all of the proteins and compounds. All solutions were prepared using ultrapure water (Millipore, Barnstead ultrapure water purification system, USA).

All electrochemical experiments were conducted using an Autolab potentiostat (PGSTAT128N, Utrecht, Netherlands) and controlled by NOVA 1.11 software. A three-electrode setup comprising indium tin oxide (ITO) glass (geometric area of 0.5 cm^2^), platinum wire, and Ag/AgCl as working, counter, and reference electrodes, respectively, was employed for all experiments. The nanostructures of PEDOTAc nanorod arrays were characterized by field-emission scanning electron microscopy (FE-SEM, ULTRA PLUS), operating at an accelerating voltage of 10 kV. X-ray photoelectron spectroscopy (XPS) spectra were acquired with a PHI 5000 VersaProbe (ULVAC-PHI, Chigasaki, Japan). Fluorescence microscopy images were captured using a confocal microscope (CQ1 Confocal Quantitative Image Cytometer CQ1, Japan), and other microscopy images were captured using a Nikon–ECLIPS Ni-E microscope (Nikon Corporation, Tokyo, Japan) and an IX81 fluorescence microscope (Olympus, Tokyo, Japan).

### OECT Device Design and Fabrication

OECT devices were fabricated on ITO-coated glass substrates (2 cm × 2 cm), where the source and drain contacts formed the active channel layers by creating PEDOTAc nanorod array films using the oxidized polymerization and followed by trans-printing methods described in the Supporting Information, based on our previously reported methods^62^ (Figure S1). The OECT active-layer channel consisted of a bottom layer of PEDOT:PSS (**PP**) and an upper layer of a PEDOTAc nanorod array (**PN**) film (Figure S2). The **PP** bottom layer, with a thickness of approximately 140 nm (Figure S3), was obtained through spin coating a PEDOT:PSS solution [containing 5 wt % DMSO and 1 wt % (3-glycidyloxypropyl)-trimethoxysilane (GOPS)] at 2000 rpm for 30 s. The **PN** upper layer was subsequently fabricated by using oxidative polymerization on a polydimethylsiloxane (PDMS) microhole array template. After polymerization, the **PN** films were thoroughly washed with methanol at least three times to remove any unreacted monomers and residual Fe(III)TOS oxidizers and IM inhibitors. The **PN** film was then transfer-printed onto the **PP** film, followed by patterning the OECT channel by using a commercial CO_2_ laser engraving system (Universal VLS 2.30, Universal System, AZ, USA) equipped with high-power density focusing optics. The channel length (*L*) and width (*W*) of the devices were 30 and 120 μm, respectively. To confine the sensing area of the **PP**/**PN**-based OECT device, a circular PDMS chamber with a volume of approximately 100 μL was employed.

### ACE2 Biofunctionalization on the OECT Active Layer

First, the **PN** active layer of the OECT device was incubated with 1× PBS solution overnight for stabilization. Then, the surface area was activated in a mixed solution of 35 mM EDC and 35 mM NHS for incubation for 30 min at room temperature. Later, the device was washed with 0.1 M PBS, and then SA solution (1 μg/mL) was added and kept for 2 h at room temperature for conjugation. Finally, the OECT active layer was immersed in ACE2 receptor solution (500 ng/mL in 1× PBS solution) for 2 h at room temperature. Afterward, the devices were washed with 1× PBS solution to remove the unreacted ACE2 solution. Then, a BSA solution (0.1%) was added for 1 h to block the remaining nonspecific binding sites of the device. Last, SARS-CoV-2 spike protein solution diluted in 1× PBS or artificial saliva was added to bind with ACE2 for a few minutes for biosensing at room temperature.

### Sample Preparation for S1 Detection in Artificial Saliva

Because the actual detection environment is much more complicated than a PBS solution, we diluted the SARS-CoV-2 spike protein in artificial saliva and incubated it on an OECT device for 10 min. The artificial saliva solution has been prepared by dissolving 0.6 g/L Na_2_HPO_4_, 0.6 g/L anhydrous CaCl_2_, 0.4 g/L KCl, 0.4 g/L NaCl, 4 g/L mucin, and 4 g/L urea in deionized water, adjusted to pH 7.4 by adding NaOH, and sterilized by autoclaving and stored at −4 °C until use.^63^ For the detection of the spike protein in saliva, a known concentration of the spike protein in 1× PBS was serially diluted with the artificial saliva solution.

All the experiments were performed with three separate measurements and recorded readings at different times with error bars (*n* = 3).

### Device Characterizations

The transfer characteristics of the OECTs were evaluated by using three-terminal electrical measurements. The integrated measurement system included two source meters (Keysight B1500A and Agilent B2912A) and a switching matrix (Agilent E5250A), all managed on a personal computer using custom LabVIEW software. The electrical signals of the as-prepared OECT device were recorded in 0.1 M PBS (pH 7.4) buffer with a Ag/AgCl wire serving as the gate electrode. The drain current (*I*_d_) was obtained by applying source-gate voltages (*V*_g_: from 0 to +0.8 V) at a fixed source-drain potential (*V*_d_ = −0.1 V). Transconductance (*g*_m_) curves were obtained by deriving the transfer curves and definition given in eq 1.
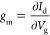
1

Following each immobilization step, the devices underwent thorough rinsing in water and subsequent testing in the electrolyte. Transfer characteristics were recorded with *V*_g_ = −0.8 to 1 V and *V*_d_ = 0.1 V. FTIR spectroscopy was conducted using the ATR-FTIR, Jason, FT/IR-6700, Tokyo, Japan. Electrochemical impedance spectroscopy and cyclic voltammetry measurements were performed with a three-electrode system utilizing an Autolab potentiostat (PGSTAT128N, ECO CHEMIE BV, Netherlands), with platinum as the counter electrode and Ag/AgCl as the reference electrode. The electrolyte used was 0.1 M PBS (pH 7.4). A SEM was used to observe the surface morphologies of the **PN** nanostructures. Before SEM examination, all of the modified surfaces were dried. To confirm the attachment of streptavidin to **PN**, the **PN** surface was incubated with streptavidin-cy5 for 1 h and observed under a confocal fluorescence microscope.

## Results and Discussion

### Characterization and Surface Morphology of PEDOT Films

**Figure 1:**
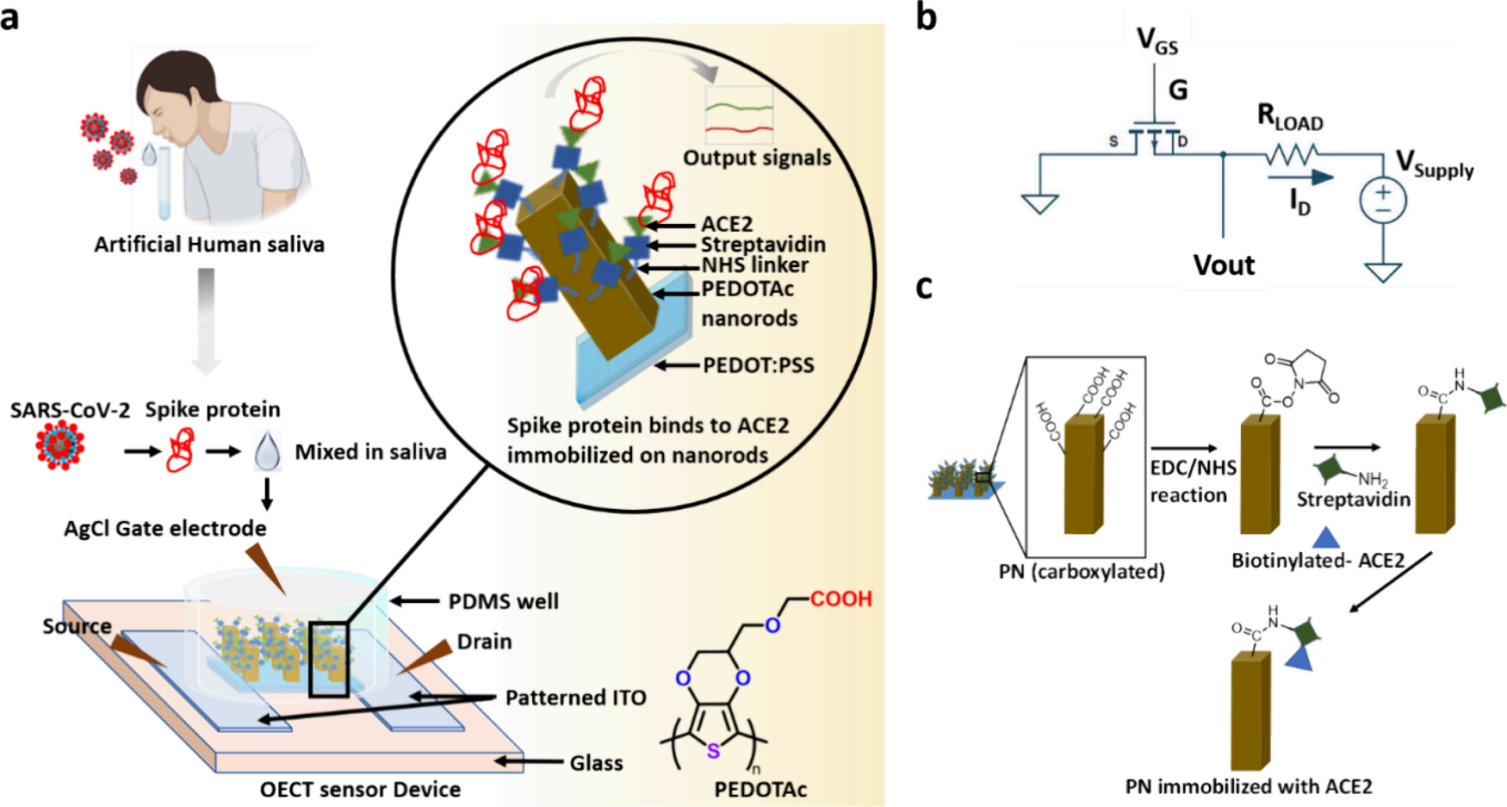
(a) Schematic of the OECT device sensing system and its saliva testing procedure for detecting the spike protein. The device features a Ag/AgCl gate electrode and PEDOT-based active-layer channel situated between the source and drain electrodes. The channel layer consists of a PN layer immobilized with ACE2 receptors. (b) Equivalent circuit diagram of the OECT. (c) Illustration of the procedure for immobilizing the biotinylated ACE2 receptor onto the streptavidin-modified PN surface, followed by the EDC/NHS reaction for streptavidin binding on PN.

We developed a surface for the detection of SARS-CoV-2 spike protein S1 using an ionized OECT device. A patterned ITO glass substrate was coated with 3D-PNs using PDMS-based transfer printing technology^62^ (Figure 1a and Figure S1). The approach for developing the modified surface is illustrated, and a step-by-step method of transfer printing, as well as fabrication of the OECT device, is given in Figures S1 and S2. The structure of a conventional OECT is illustrated in Figure 1b. Conducting polymer poly(3,4-ethylenedioxythiophene)-carboxylated fabricated on poly(styrenesulfonate) (PEDOT:PSS) was employed as the channel layer and functionalized with an ACE2 receptor using EDC/NHS reaction as shown in Figure 1c. This method involves the specific noncovalent coupling of biotinylated ACE2 to SA-modified **PP**/**PN**. Amine coupling was used for the covalent attachment of streptavidin on carboxyl groups of the PN. SA was attached covalently to acid-treated **PN** via EDC/NHS coupling to yield stable amide linkages. Subsequently, ACE2-biotin was allowed to bind specifically to the streptavidin.

The EDC/NHS coupling reaction used here ensures the strong immobilization of streptavidin on engineered PEDOTAc nanostructures, and biotin–streptavidin interaction allows high-affinity, noncovalent attachment of ACE2. This will also ensure its stability and functionality over multiple washing steps or prolonged usage^64,65^ (Figure 1c).

**Figure 2:**
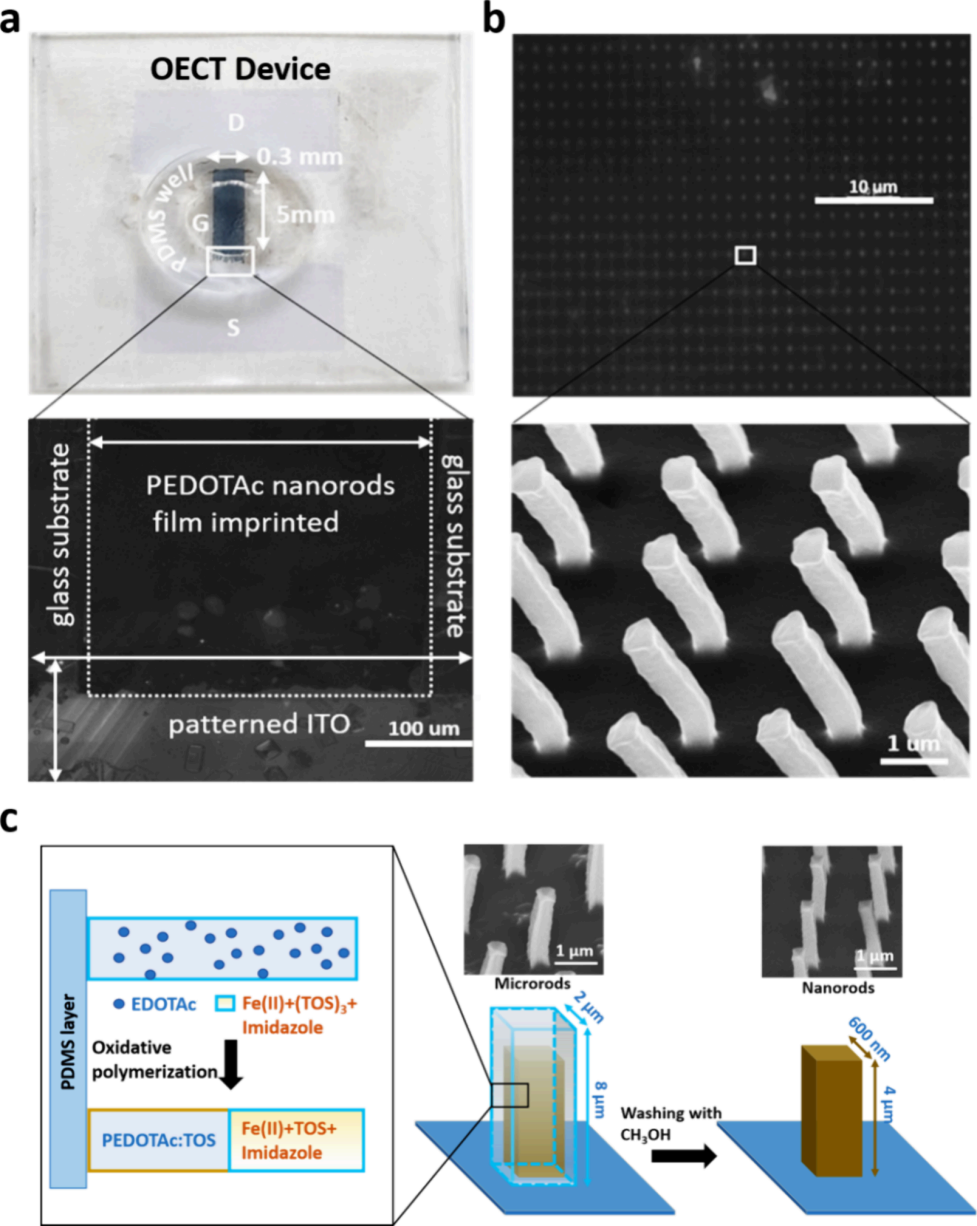
(a) Photograph (top) and optical microscope image (bottom) of a PEDOTAc nanorod array-based OECT biosensor, depicting a cylindrical PDMS chamber on patterned ITO. (b) PN array visible under an optical microscope (top) with a magnified view under SEM (bottom). (c) Schematic representation of the polymerization of the PEDOTAc precursor, illustrating the two-phase segregation of PEDOTAc and TOS within the negative PDMS hole array replicate.

Consistent with previous studies,^62,66^ SEM images (Figures 2a,b) confirm the **PN** morphology, showing an average width, height, and underlying layer thicknesses of approximately 750 nm, 5 μm, and 650 nm, respectively, with a 4 μm spacing between nanorods, as shown in Figure S3. The **PN** surface was used for linking receptors to other molecules. The unique shape of **PN** enhances the surface area and sensitivity of the nanorods, making them ideal for immobilizing receptors in detection experiments. Figure 2c presents an SEM image of 3D PN-based rod arrays showing rod sizes that were transferred to ITO glass without undergoing a methanol washing procedure. On the left side, the rods have a width of approximately 1 μm, matching the size of the Si microrod arrays, while the right side features rods with proper nanorod dimensions. This discrepancy may result from chemical oxidative polymerization on the negative PDMS replicate, leading to the formation of **PN**-TOS microrod arrays. These arrays consist of a **PN**-based nanorod core surrounded by a thick TOS layer. To confirm this structure, the TOS coatings were removed from the **PN**-TOS microrod arrays by washing them with methanol three times, resulting in 3D PN-based nanorod arrays. Table 1 shows that positive Si masters (Si-8) with microrods of height 8 μm, width 2 μm, and period 4 μm allowed us to efficiently produce PEDOT-based nanorod arrays (width: 0.6–0.7 μm, height: 3–4 μm, period: 4 μm) in comparison to a flat structure.

**Table 1:** Geometric Parameters of the Si Microrod Array Master and PEDOTAc-Based Nanorod Array on the Ionized OECT Devices

device	height (H, μm)	width (W, μm)	period (P, μm)	aspect ratio	replication fidelity (%)
master Si-8	8	2	4	4	
S-1	3.5	0.750	4	4.6	100
S-2	4.2	0.782	4	5.37	100
S-3	3.5	0.690	4	5.07	100

### Immobilization of the ACE2 Receptor on the PP/PN Film

This method involves the noncovalent attachment of biotinylated ACE2 to the SA-modified **PP**/**PN** film. The EDC/NHS coupling approach was employed for the covalent binding of SA to the carboxyl groups on the **PP**/**PN** surface (Figure 1c). Subsequently, the biotinylated ACE2 receptor was attached to SA due to the SA–biotin interaction, known for being the strongest noncovalent affinity interaction. This makes the SA–biotin reaction highly suitable for the following sensor development applications.

### Characterization with FTIR, Water Contact Angle, XPS, and Confocal Microscopy

**Figure 3:**
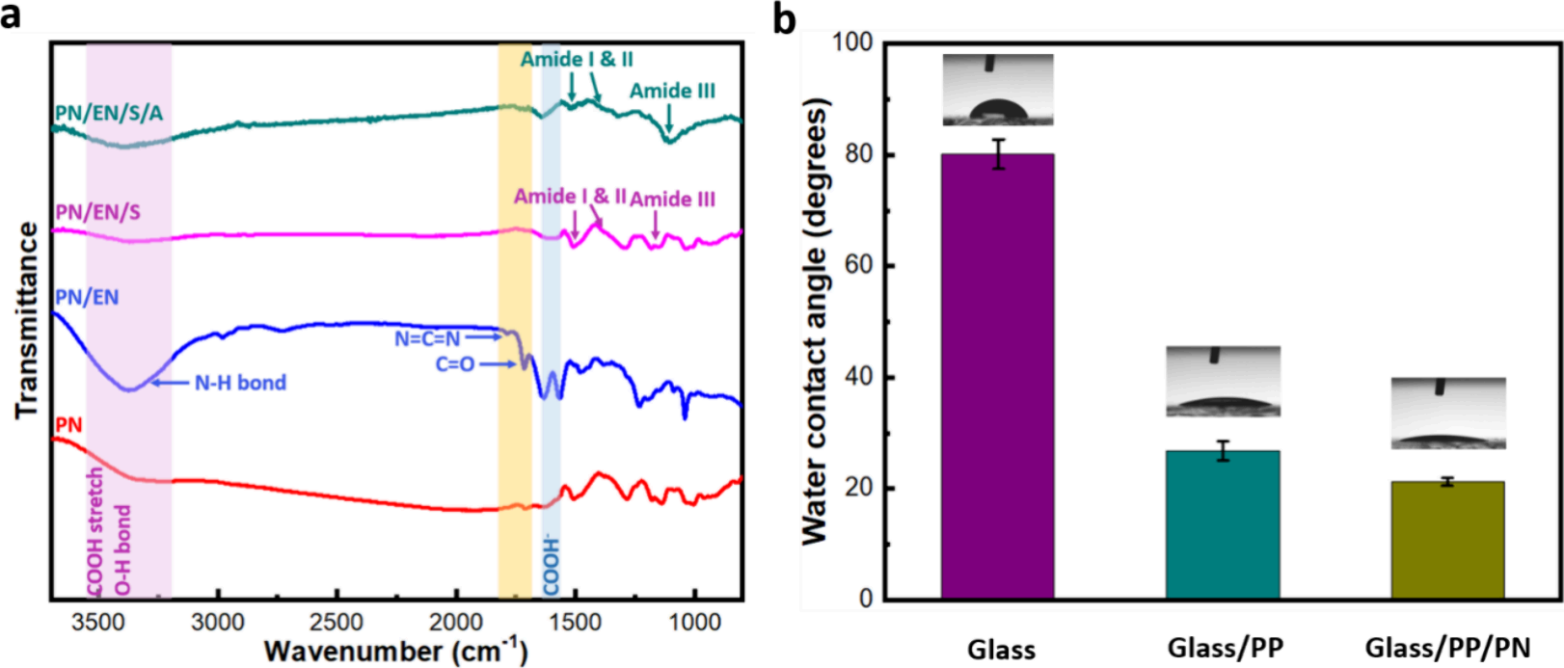
(a) FTIR spectra showing the modifications of PN through the EDC/NHS reaction, followed by streptavidin and ACE2 receptor binding. (b) WCA measurements of Glass, Glass/PEDOT:PSS, and Glass/PEDOT:PSS/PEDOTAc. Error bars were obtained by taking average of *n* = 3 separate measurements taken at different times.

Fourier transform infrared (FTIR) and X-ray photoelectron spectroscopy (XPS) were used to characterize the PN morphology and surface/elemental composition. XPS was used to analyze the chemical composition of the surfaces of the I (black), I/PP (red), I/PP/PN (blue), I/PP/PN/E (green), I/PP/PN/E/S (violet), and I/PP/PN/E/S/A (orange) as shown in Figure S4a. There is a high sulfur peak present in the XPS spectrum of I/PP/PN in comparison to bare ITO. A high-resolution XPS spectrum of the I/PP/PN reveals two characteristic peaks at 226.8 and 162.4 eV, which correspond to the binding energies of S_2s_ and S_2p_, respectively. These results confirmed that PEDOTAc is successfully functionalized on the surface of ITO. Meanwhile, surface analysis of I/PN glass reveals the absence of Sn and In peaks, which are characteristic chemical structures of ITO glass. Figure S4b shows the chemical composition of each element present on the surface based on the XPS spectra. The successful immobilization of the protein on the OECT active-layer channel surface is also confirmed by FTIR spectroscopy measurement performed before and after EDC/NHS and ACE2 immobilization (Figure 3a). A broad peak of −COOH was found at 3400 cm^–1^ corresponding to the carboxyl group of PEDOTAc. An OH stretch was also observed at 3733 cm^–1^. After the EDC/NHS amine coupling reaction, a C=O stretch was observed at 1777 cm^–1^ and a small peak was observed around 1918 cm^–1^, suggesting the presence of the N=C=N functional group from EDC. Notably, the N=C=N peak disappeared after subsequent functionalization with streptavidin and ACE2. Here, two distinct stretches around 1657 and 1548 cm^–1^ were visible after the immobilization of these proteins. The two bands correspond to the amide I (1600–1690 cm^–1^) and amide II (1480–1575 cm^–1^) bands, which represent two prominent stretches of the protein backbone, were observed. The amide I band represents the C=O stretching vibrations of the peptide bonds, and the amide II band represents the N–H and C–N stretching bonds. Two prominent vibrational bands of the protein backbone can be observed after the immobilization of the antigen and antibody. Taken together, these results confirm that the modification process was successful.

Surface wettability analysis is performed to examine the degree of polymer fabrication and the nature of the surface before and after polymer attachment. In this study, we recorded and quantified water contact angles (WCAs) for Glass, Glass/PP, and Glass/**PP**/**PN**. Glass surfaces are used as the control (Figure 3b). Glass surface exhibits an obvious hydrophobicity with a WCA of 80°. After PP coating, WCA decreases significantly to 26.8°, which verifies an effective coating. Importantly, a further substantial decrement in the WCA (21.2°) is observed after **PN** functionalization, which indicates that the surface is hydrophilic. Noted that hydrophilic surfaces interact readily with water, which is crucial in enhancing a sensor’s ability to detect and measure substances in the aqueous environment. A hydrophilic surface can greatly facilitate the immobilization of biological molecules thus improving sensor performance by ensuring better adhesion. Additionally, these surfaces can effectively reduce undesired, nonspecific binding and fouling by proteins and other biomolecules, maintaining accuracy and prolonging sensor life.^67^

**Figure 4:**
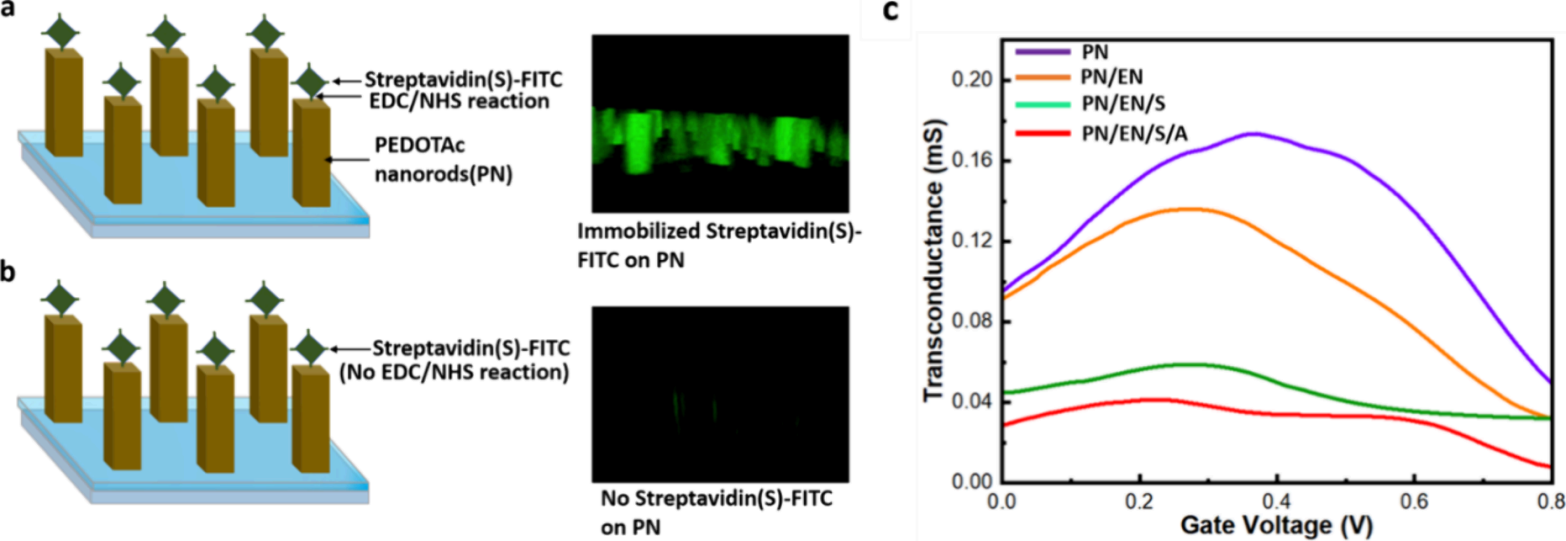
(a, b) Confocal microscopy images confirming the attachment of streptavidin. (a) Fluorescence is visible on the nanorod arrays after the modification of streptavidin-cy5 on the EDC/NHS-treated surface. (b) No EDC/NHS reaction on the surface. (c) Transconductance (*g*_m_) of the OECT device as a function of active-layer channel, showing the response with the addition of various linkers for ACE2 antibody immobilization.

Following previous steps, the **PN** surface was treated with streptavidin-cy5 for 15 min to confirm the successful attachment of the streptavidin. A confocal microscope was used to examine the treated surface. Figure 4a,b shows the fluorescence images of visible nanorods, confirming the successful modification of nanorods with streptavidin. Figure 4c and Figure S5 display the transconductance and transfer curve, respectively, under a negative sweeping bias of drain voltage (*I*_d_ from 0 to −1.0 V) for OECT devices, which have been functionalized with active channel layers with PN over PP on ITO glass with different linkers added for the ACE2 receptor immobilization. As shown in Figure 4c, the transconductance (*g*) measurements of the OECT device for PN (violet line), PN/EN (orange line), PN/EN/S (green line), and PN/EN/S/A (red line) demonstrate a decrease in transconductance with each subsequent surface modification.

### Electrochemical Properties of PEDOT Films

The modification steps with nanorods using the trans-printing method were followed by Impedance spectroscopy analysis to see how the surface behaved electrochemically. The electrochemical response was recorded by using 0.1 M PBS. Figure S6a shows the CV response and change in the current peak following the PP and PN functionalization on the glass surface. The current response improved after the PN layer formation, as it enhanced electron transfer efficiency. A combined effect of PN and their nanostructures is that they enhance the current response and increase the sensitivity. Also, the impedance response of the layer was recorded before and after PN fabrication on PP. The impedance was recorded at frequencies of 0.1–10,000 Hz and 0.25 V. The resistance was shown to be decreased after PN was fabricated (Figure S6b).

### Output Characteristics and Transfer Curves of the OECT Device

Transfer curves, which plot the drain current as a function of gate voltage in the OECTs, were recorded after each surface modification step of the surface, including the immobilization of ACE2 within the channel area using an EDC/NHS coupling reaction as mentioned earlier (Figure S5). The electrolyte used for testing was a 0.1 M PBS solution with a pH value of 7.4, commonly regarded as the standard testing environment. In this process, the binding between S1 and the receptor was translated into a readable electrical signal by the OECT device, serving as a signal transducer in the biosensor system.

The attachment of ACE2 to the active channel layer of the OECT device was validated by analyzing the transfer curves and output characteristics of the device before and after receptor immobilization. As shown in Figure 4c and Figure S5, both the drain current and transconductance (0.1 M PBS, pH 7.4) of the modified OECT device decreased following the EDC/NHS reaction, as well as streptavidin and ACE2 immobilization onto the active layer. This observation aligns with the formation of an insulating layer in the active channel region due to the binding of streptavidin and ACE2 antibody, which reduced the mobility of major charge carriers from the electrolytes to the channel. These measurements confirm the successful immobilization of the ACE2 in the active area of the OECT device.

### Response of the OECT Device to the Addition of the S1 Protein

**Figure 5:**
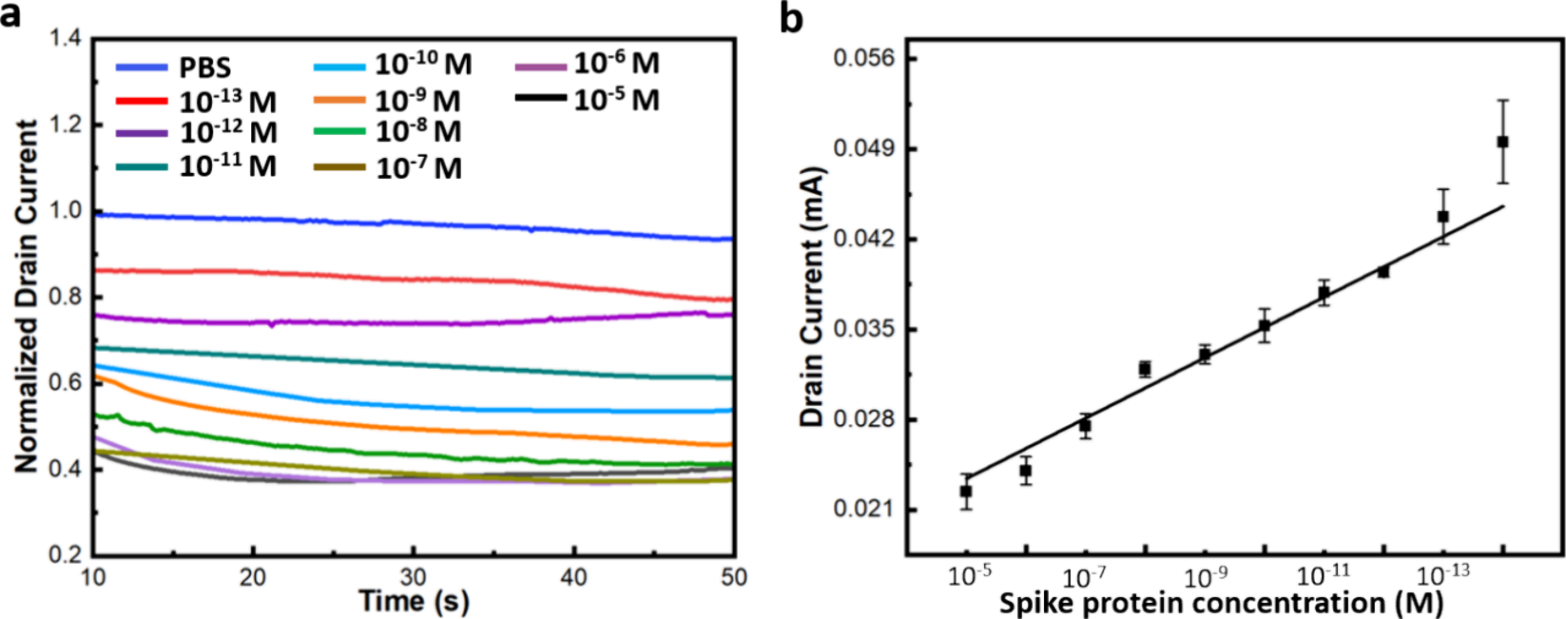
(a) Sensitivity analysis of the device targeting the S1 protein. (b) Plot showing the change in the peak of drain current response versus S1 protein concentration. Each data point represents the average of three separate experiments (*n* = 3), with error bars indicating the standard deviation.

The detection process of the S1 protein involves three sequential steps: (i) incubating the S1 protein on the ACE2-modified OECT channel, (ii) washing the channel with PBS buffer to remove unbound S1 and any physically absorbed biomolecules, and (iii) characterizing the device performance in an electrolyte. Since rapid detection is crucial for diagnosing target viruses in large-scale infections, we conducted a real-time investigation of the response of the OECT for the selective detection of the S1 protein. S1 solutions at concentrations ranging from 10^–5^ to 10^–13^ M in PBS (0.1 M, pH 7.4) were incubated on the OECT device, allowing them to bind to the channel area. Figure 5a depicts the real-time amperometric response, representing the normalized drain current (*I*_d_ vs time) at varying concentrations of the S1 protein in PBS, with a gate voltage (*V*_g_) of 0 V and a drain bias (*V*_d_) of −0.4 V. After stabilizing the device with PBS, specific S1 concentrations were introduced into the PDMS well. The sensor demonstrated a linear response over a wide range of S1 concentrations, indicating strong sensitivity to the target protein. The calibration curve exhibits excellent linearity with an *R*^2^ value of 0.97 (Figure 5b), based on the drain current–time response of the device at different S1 concentrations (*n* = 3), ranging from 10^–13^ to 10^–5^ M in PBS (0.1M, pH 7.4). The equation for the linear plot is expressed as *y* = 0.00280*x* + 0.01906. The Limit of Detection (LOD) was demonstrated to be 138 fM, taking into account the molecular weight and molarity of the target spike protein. These results confirm the device’s strong potential for sensitive detection capabilities. As the concentration of the SARS-CoV-2 spike increases, the transfer curves gradually shift. The plausible explanation for this is that proteins are zwitterionic and consist of amino acids, which may have positive or negative charges depending on the isoelectric point (pI) and pH of the electrolyte.^68^ The SARS-CoV-2 spike protein carries a negative charge in PBS solution (pH 7.4), as it is negatively charged at pH values above 6.24.

When the target spike protein binds to the receptor immobilized on the channel, it introduces charge or dipole effects near the channel surface, which changes the channel’s electrical properties. Notably, the 3D-OECT device with PEDOTAc nanorod arrays offers a significantly larger number of reaction sites, attributed to its larger surface area, for binding with the target protein, compared to the conventional planar design of the OECT channel layer. Since proteins are charged molecules, and when they bind to the receptor (ACE2), they change the local distribution of charges near the channel. These charges interfere with the electrostatic gating effect and affect the capacitance (*C*_i_) of the OECT. This alters the effective gating efficiency, thereby modulating *V*_gs_ and, consequently, *I*_ds_. This enhanced interaction results in a stronger electrical response, making the device highly effective for biosensing applications. In this case, the charges from the bound protein may screen the charge carriers (holes in the PEDOTAc layer), reducing their mobility.^69^ This leads to a reduction in channel conductivity and an increase in resistance, leading to the observed decrease in *I*_ds_ as shown in Figure 5a and Figure S7. The negative charge on the protein may attract charged ions from the electrolyte, altering the local charge distribution at the channel surface.

The channel current (*I*_ds_) of an OECT is described by eq 2.
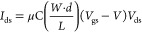
2

*I*_ds_ is the drain-source current, μ is the charge carrier mobility, C is the capacitance per unit area of the dielectric layer, *W* is the channel width, *L* is the channel length, *d* is the channel thickness, *V*_gs_ is the gate-source voltage, *V* is the threshold voltage, and *V*_ds_ is the drain-source voltage.

The excellent sensitivity of the developed sensing device can be explained by 3D PN (three-dimensional nanorod) nanostructures, which offer several advantages over other nanostructured surfaces, such as nanowires or nanoparticles, for electrochemical sensing and receptor immobilization. First, the vertically aligned nanorods create a high surface area-to-volume ratio, which gives more active sites for receptor immobilization and enhances the density of the ACE2 receptors. Compared with nanoparticles, the nanorods facilitate efficient charge transport, enabling faster signal transduction. Additionally, the 3D nanostructure allows for better electrolyte diffusion, which is particularly beneficial for detecting low concentrations of target proteins. The functionalization of the nanorods with ACE2 receptors via EDC/NHS chemistry ensures strong covalent bonding, enhancing stability and selectivity during sensing. Together, these features make the 3D PN structure more effective for electrochemical sensing applications and faster response times compared to the conventional planar design of the channel layer in OECTs.^70^

### SARS-CoV-2 Spike Protein Detection in Artificial Saliva Samples

**Figure 6:**
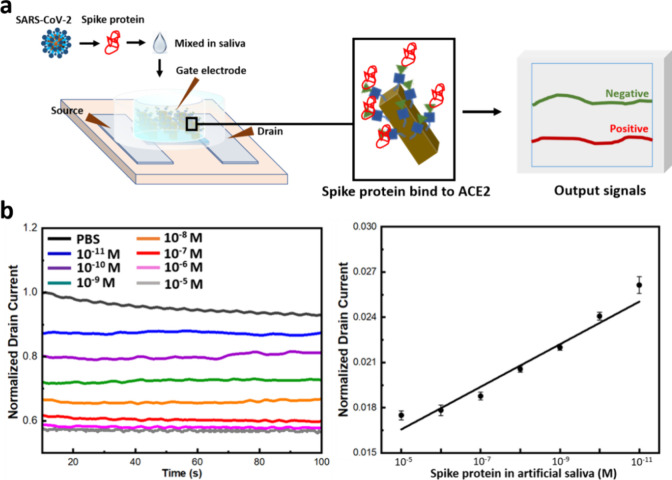
(a) Schematic of the sensitivity experiment using the amperometric response in an artificial saliva solution of 0.1 M PBS (pH 7.4) buffer. (b) Normalized *I*_ds_–time response for different concentrations of spike S1 protein, ranging from 10^–5^ to 10^–11^ M. A calibration curve with standard deviations (*n* = 3) obtained from the *I*_ds_–time response of the OECT device for varying spike protein concentrations within the same range. Each data point represents the average of three separate experiments (*n* = 3), with error bars indicating the standard deviation.

It is known that the actual detection environment is more complicated than a PBS solution; we thus tested spike protein in artificial saliva prepared and incubated it on devices for 5 min as shown in Figure 6a. Following the incubation of the S1 protein diluted in saliva, the amperometric response was recorded (Figure 6b). The protein was diluted in the range of 10^–5^ to 10^–11^ M in artificial saliva for detection. The LOD of the device for measuring the spike protein in saliva samples is determined to be 10 pM, which is higher than the LOD in PBS (138 fM), indicating reduced sensitivity in saliva due to the presence of other proteins that can cause nonspecific adsorption or interfere with the detection signal.

However, SARS-CoV-2 spike protein detections in saliva exhibit a good linear relationship within their concentration ranges (Figure 6b). The detection range in serum ranges from 10 pM to 1000 nM. The calibration curve in Figure 6b shows good linearity (*R*^2^ = 0.95) based on the drain current–time response of the OECT device with standard deviations (*n* = 3) when different spike protein concentrations ranging from 10^–5^ to 10^–11^ M in PBS (1×, pH 7.4) were introduced. The regression equation for the linear plot is expressed as *I*_d_ = 0.00141*x* + 0.01515. The results yielded an LOD of 107 pM, which is sufficient for detecting low concentrations of spike proteins in saliva. Therefore, the high sensitivity of our developed OECTs meets the necessary criteria for detecting spike protein levels in saliva, confirming their potential for COVID-19 virus protein detection.

Additionally, due to the conserved nature of the ACE2 target protein binding domain among different variants, we expect that the developed device will show a similar response. This assumption is supported by prior studies that have shown ACE2 binding to be largely preserved across key variants.^71^

### Specificity and Stability Testing

**Figure 7:**
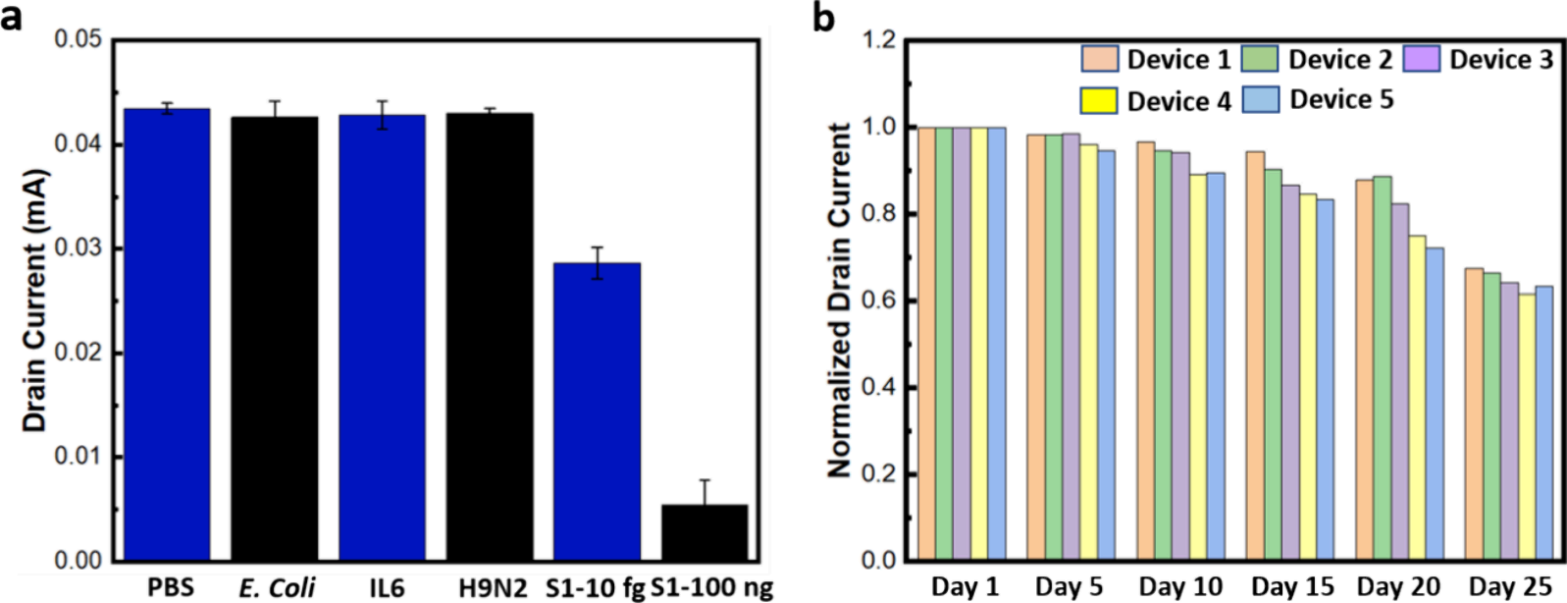
(a) Selectivity of the developed OECT device against various nonspecific proteins, including H9N2, IL6, and *E. coli*. (b) Stability test results showing the normalized *I*_ds_–time response of the OECT device over 20 days. Error bars were obtained by taking average of *n* = 3 separate measurements taken at different times.

The isolation of the *E. coli* protein was performed using the method given in the Supporting Information. To validate the specificity of the sensor in detecting SARS-CoV-2 S1, the spike protein was tested under the same conditions (Figure 7). We prepared solutions containing various interfering proteins at a concentration of 100 ng/mL and target proteins at concentrations of 10 fg/mL and 100 ng/mL. The transfer curve of the device exhibited negligible shifts after incubation with various nonspecific proteins, such as H9N2, IL6, and *E. coli*, on the PN-modified channel layer. In contrast, a significant shift was observed when the mixture was incubated with the spike protein under identical conditions. Additionally, incubating the sensor in PBS elicited no response in the transfer curve, indicating the excellent specificity of the device in aqueous solutions. The Output characteristics, transfer curve, and transconductance graphs are shown in Figures S8a–h.

The 3D-PN-based OECT device short-term stability test was performed (Figure S9). The results show good stability over 1000 cycles with a pulse interval time of 1s and an *I*_d_ of −0.4 V). Furthermore, the reproducibility of the OECT device sensor was tested from the *I*_d_–time response using 50 ng/mL spike protein incubation with three fabricated OECT devices. The OECT sensor devices showed high reproducibility. The stability of the OECT device was further investigated from *I*_d_–time response at a gate voltage pulse of 0.1 V (vs Ag/AgCl) for 1000 cycles with a pulse interval time of 1 s and drain voltage −0.4 V. The *I*_d_ response did not show any significant changes with time, after 1000 cycles, indicating that the **PN** layer over the OECT device is highly stable without any degradation. The long-term stability of the ACE2 functionalized device was tested for more than 20 days and was found to be stable for up to 20 days in PBS as shown in Figure 7b. The observed issue may stem from the hygroscopic nature of PEDOTAc, which absorbs moisture from the environment. This moisture can lead to delamination or swelling of the underlying PEDOT:PSS film when it interacts with the electrolyte under long-term stability tests. Such changes disrupt charge transport pathways, ultimately causing a decrease in conductivity.^72^

The process for the fabrication relies on easily available ITO substrates and the minimum use of PEDOT polymers, which are relatively low-cost and widely used in the electronics industry. Simultaneously, using a CO_2_ laser to define the channel area is highly advantageous due to its precision, speed, and low operational costs, enabling the rapid design of the nanoelectrode surface. Meanwhile, functionalization with ACE2 receptors via EDC/NHS chemistry is a well-established method. The whole process usually takes a maximum of 1 day for device production. Despite these advantages, there are challenges in scalability for batch fabrication of uniform nanorods using the trans-printing method, which we plan to address in our future study.

## Conclusions

In summary, we successfully developed a portable, label-free, and highly sensitive 3D-OECT device utilizing PEDOTAc nanorod arrays and PEDOT:PSS as the upper and bottom layers, respectively, of the **PP**/**PN**-based active-layer channel for the rapid detection of the SARS-CoV-2 spike protein in artificial saliva. The three-dimensional OECT channel layers were surface-modified with ACE2 receptors using EDC-NHS and SA–biotin approaches, enabling selective sensing of spike S1 proteins through a specific antibody–antigen reaction. This 3D-OECT device achieved an LOD of 138 fM in PBS solutions and 107 pM in artificial saliva, demonstrating sensitivity comparable to those of many existing detection methods (Supporting Information and Table S1). Notably, the detectable range includes concentrations of spike S1 protein typically found in artificial saliva, underscoring the promising potential for practical biosensing applications. Moreover, by applying voltage pulses to the OECT gates during the S1 protein incubation, the targeted protein was detected more rapidly compared to incubation without electrical stimulation, with stable signals obtained within just 2 min. We believe that the device sensitivity can be further enhanced by addressing the challenges associated with the fabrication of uniform and stable nanorods, which will be a focus of future studies. Additionally, we plan to design the device to enable the detection of multiple targets simultaneously. We anticipate that this biosensor platform can be adapted for the rapid detection of viruses associated with various other diseases.

## Supporting Information Available

Fabrication process of PEDOTAc-based 3D-nanorod arrays and its OECT device, cross-sectional SEM image of the OECT channel layer, XPS analysis of the OECT channel layer, device characterization of the PN-based OECT sensor, electrochemical properties of PP- and PN-coated ITO electrodes, interference and long-term stability study, and device measurement and on–off mechanism of the device (DOCX)
