# Supplementary material for: Poly(3,4-ethylenedioxythiophene) Nanorod Arrays-Based Organic Electrochemical Transistor for SARS-CoV-2 Spike Protein Detection in Artificial Saliva
Source: ACS Sens. 2025 Mar 13;10(3):2007–18. doi: 10.1021/acssensors.4c03207 (PMC11959606; doi:10.1021/acssensors.4c03207)
Supplement: Supplementary file 1 — se4c03207_si_001.docx [file se4c03207_si_001.docx]

Supporting Information

**Poly(3,4-ethylenedioxythiophene) Nanorod Arrays-based Organic Electrochemical Transistor for SARS-CoV-2 Spike Protein Detection in Artificial Saliva**

Syed Atif Ali,^a,b,c,d,e^ Ying-Lin Chen,^a^ Hsueh-Sheng Tseng,^a^ Hailemichael Ayalew,^b,c^ Jia-Wei She,^b,e^ Bhaskarchand Gautam,^e^ Hsiung-Lin Tu,^c*^ Yu-Sheng Hsiao,^a*^ Hsiao-hua Yu^b,c,d*^

^a^Department of Materials Science and Engineering, National Taiwan University of Science and Technology, Taipei 106335, Taiwan

^b^Smart Organic Materials Laboratory, Institute of Chemistry, Academia Sinica, Nankang, Taipei 11529, Taiwan

^c^Institute of Chemistry, Academia Sinica, Nankang, Taipei 11529, Taiwan

^d^Sustainable Chemical Science & Technology, Taiwan International Graduate Program (TIGP), Academia Sinica, Nankang, Taipei 11529, Taiwan

^e^Department of Applied Chemistry, National Yang Ming Chiao Tung University, Hsinchu 30010, Taiwan

^f^Department of Engineering and System Science, National Tsing Hua University, Hsinchu 30010, Taiwan

*Corresponding authors at:

Dr. Hsiung-Lin Tu: Institute of Chemistry, Academia Sinica, Nankang, Taipei 11529, Taiwan; E-mail: [*hltu@gate.sinica.edu.tw*](mailto:hltu@gate.sinica.edu.tw)

Dr. Yu-Sheng Hsiao: Department of Materials Science and Engineering, National Taiwan University of Science and Technology, Taipei 106335, Taiwan; E-mail: [*yshsiao@mail.ntust.edu.tw*](mailto:yshsiao@mail.ntust.edu.tw)

Dr. Hsiao-hua Yu: Institute of Chemistry, Academia Sinica, Nankang, Taipei 11529, Taiwan; [*bruceyu@gate.sinica.edu.tw*](mailto:bruceyu@gate.sinica.edu.tw)

**Experimental Section**

**Fabrication of carboxyl-functionalized poly(3,4-ethylenedioxythiophene) (PEDOTAc) based 3D-nanorod arrays (PNs)**.

The negative PDMS transfer printing process has been previously described in detail.^S1^ A nanorod pattern fabricated on a silicon wafer using photolithography to create silicon masters was obtained from Prof. Yu-Sheng Hsiao, Department of Material Science and Engineering, NTUST. The PDMS base (Sol A) and curing agent (Sol B) (Sylgard 184, Dow Corning) in a 10:1 (w/w) ratio were mixed to create a negative PDMS template. After mixing, the solution was poured onto the Si-microrod array master, and air bubbles were removed by placing it in a desiccator. The curing was performed at 60°C for 3 hours in an incubator. Once cured, the negative PDMS nanorod array replicates were used in the transfer printing process to transfer the nanorod structures onto ITO glass as outlined in **Figures S1**a−g.

PNs were fabricated via chemical oxidative polymerization of EDOTAc. Prior to experimentation, patterned ITO-coated glass substrates were sequentially cleaned with detergent, de-ionized water, acetone, methanol, isopropanol, and water, followed by ultrasonication for 20 minutes at each step. After washing, the patterned ITO glasses were dried using nitrogen gas.

**
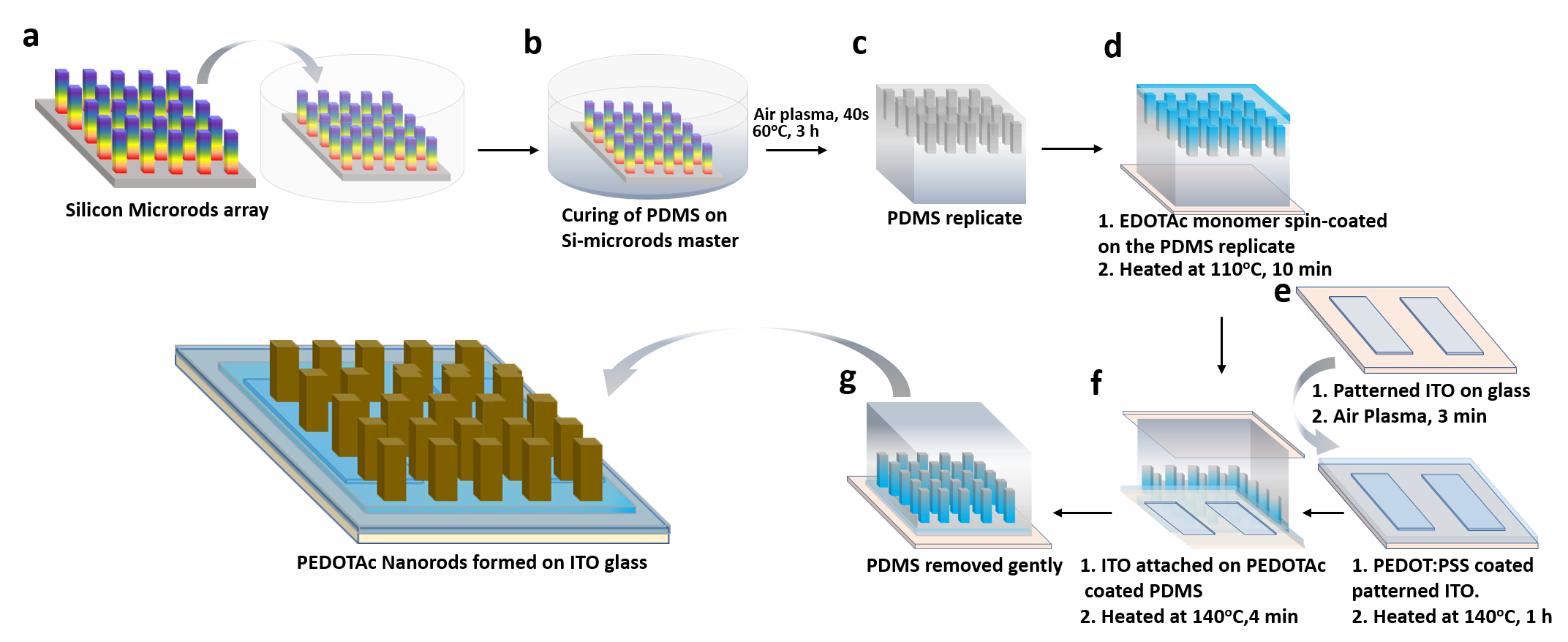
**

**Figure S1.** 3D-nanorod PEDOTAc array process of fabrication on the ITO glass. (a−c) PDMS replicates the Si microrod surface preparation process. (d) After treatment with air plasma PEDOTAc spin-coated on the PDMS replicate and heated for chemical oxidative polymerization. (e−f) ITO was functionalized with APTS using a hotplate. (g) Polymerized PN on PDMS was attached to the ITO glass. ITO glass was detached gently from PDMS for PN arrays.

Imidazole solution (1.47 M) and a Fe(III)TOS solution (2.56 M), an oxidizing agent, were both prepared in methanol as solvent. The EDOTAc monomer solution was prepared by combining optimized concentration of 154 mM EDOTAc with an equal volume of 1.8 mL of Imidazole and Fe(III)TOS solutions. The PDMS template was then treated with air plasma (10 mTorr) (Harrick Plasma, PDC-32G) for 40 sec. To initiate chemical oxidative polymerization, the mixture was spin-coated onto the air plasma-treated PDMS template at 2000 rpm for 10 seconds. The PDMS template was then placed on a hotplate at 110°C for 10 minutes for polymerization. Polymerization of the EDOTAc coating on the PDMS surface proceeded for 6 minutes, resulting in a color change from yellow to dark green. This process was repeated four to five times. Subsequently, the coated film was cooled to room temperature and washed with methanol to remove any unreacted monomers and excess Fe(III)TOS.

To transfer the synthesized PN, a layer of 3-aminopropyltrimethoxysilane (APTS) was first applied to the patterned ITO on the glass substrate (**Figure S2**a), followed by a PEDOT:PSS coating to enhance adhesion between the PN and the substrate (**Figure S2**b). Next, a PDMS film coated with the synthesized PEDOTAc nanorod array film was brought into contact with the PEDOT:PSS-coated ITO substrate. The PDMS film was gently peeled off, leaving the transferred nanorods on the patterned ITO surface (**Figure S2**c). The ITO substrate with the transferred nanorods was subsequently baked at 140°C for 1 h on a hotplate to improve adhesion and remove any residual solvent. The fabricated surface was then patterned using a CO_2_ laser to define the active channel area (**Figure S2**d). Afterward, ACE2 receptors were immobilized onto the channel via an EDC/NHS reaction, transforming it into a functional OECT sensing device (**Figure S2**e). Finally, the device was encapsulated with a PDMS well to prepare it for subsequent sensing experiments (**Figure S2**f).


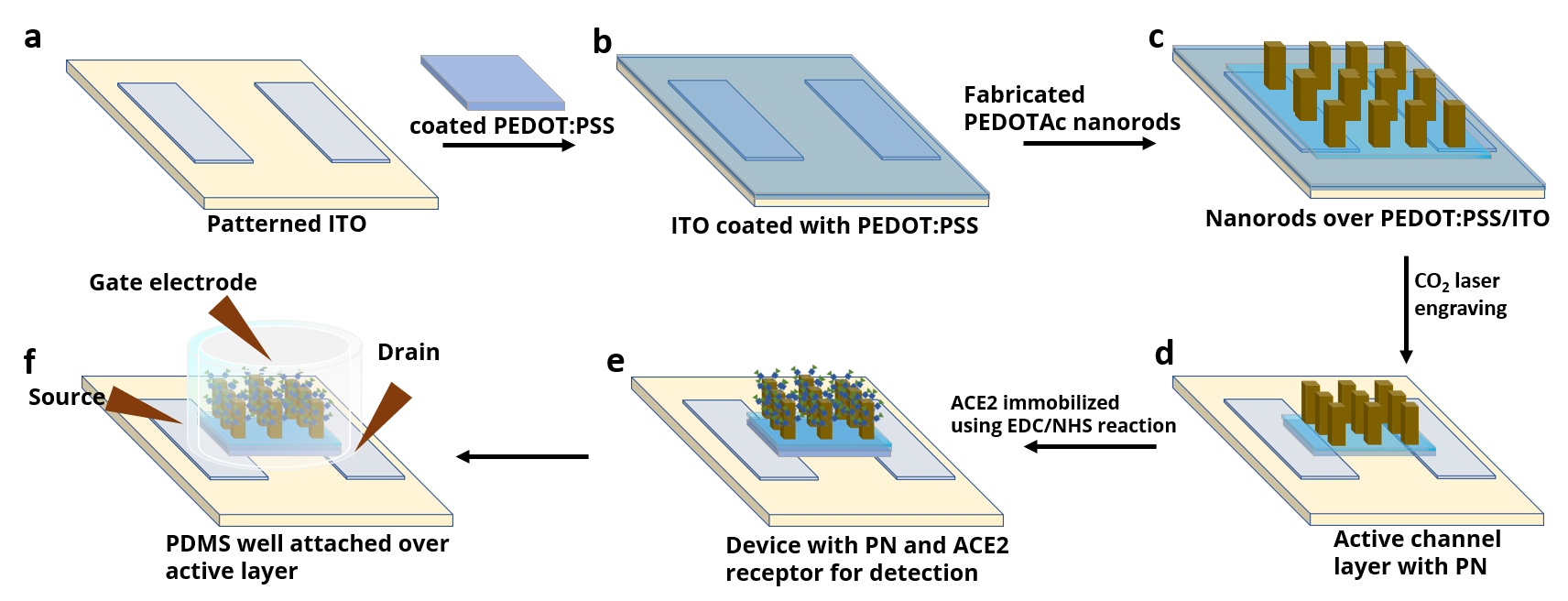


**Figure S2.** Stepwise fabrication process of the OECT sensing device: (a) Cleaned patterned ITO substrate, (b) PEDOT:PSS coating on the patterned ITO, (c) Transfer of PN onto the PEDOT:PSS/ITO surface using transimprinting technology, (d) Patterning of the active channel layer using a CO_2_ laser, (e) Functionalization of the modified active layer with ACE2 via EDC/NHS reaction, and (f) Final OECT sensing device encapsulated with a PDMS well for sample detection.

**Preparation of target and non-target proteins**

The bacterial whole protein was isolated from *E. coli* following a previously reported protocol. Initially, the bacteria were thawed and cultured in lysogenic broth (LB) and incubated on a shaker at 37 °C for 24 hours. After incubation, 150 μL (1 × 10⁷ CFU/mL) of the thawed bacterial culture was spread onto a nutritive agar plate using the spread plate method. The following day, a bacterial colony was transferred into 5 mL of LB broth and grown overnight at 37 °C. The resulting bacterial culture was then used for isolating the whole protein, which served as a non-specific protein target for sensing applications. The non-specific proteins used in the study, H9N2 and IL6, were purchased from Sinobiological (China).


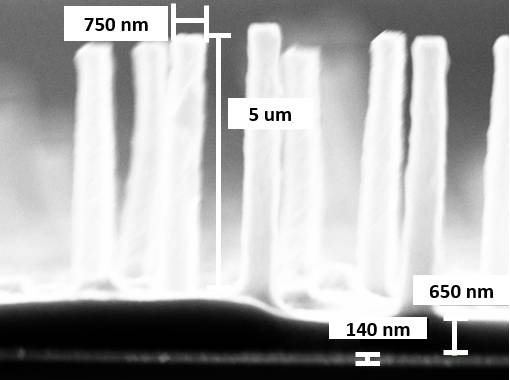


**Figure S3.** Cross-sectional SEM image of OECT channel layer. Scale bar: 1μm.


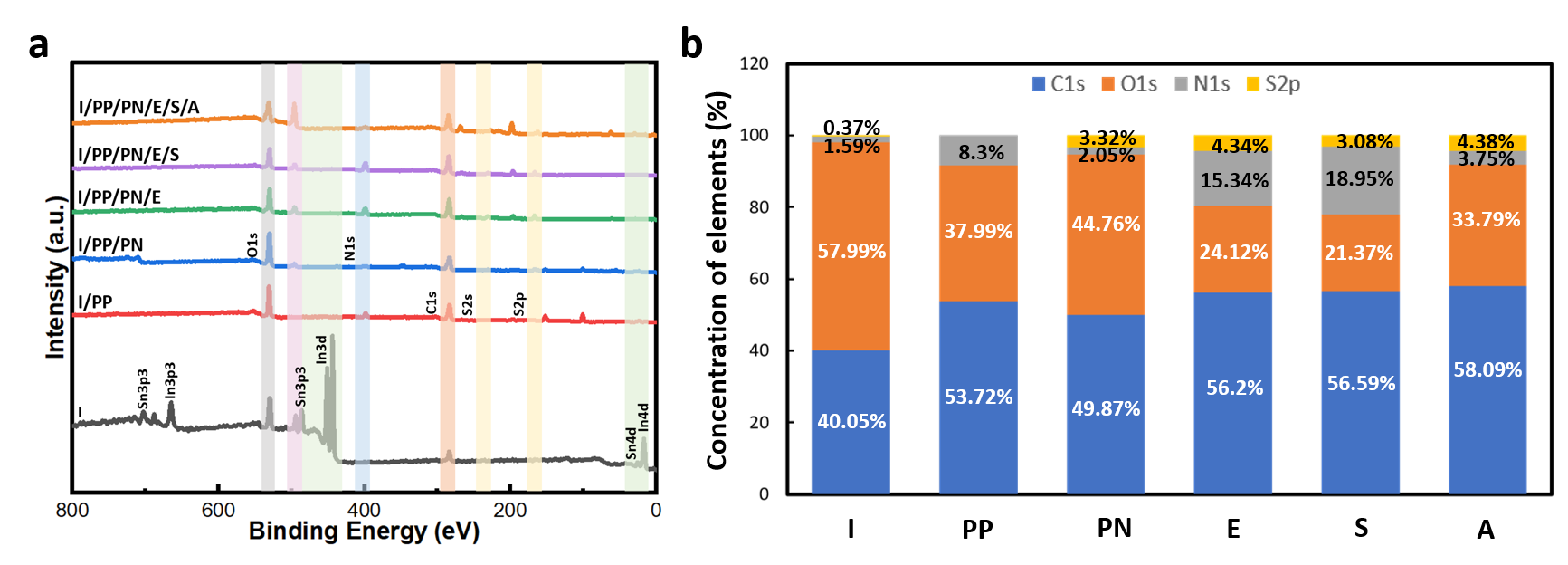


**Figure S4.** (a) XPS analysis of OECT channel layers (b) Elemental analysis (atomic percentage) of OECT channel layers based on the XPS results. Scale bar: 1μm.


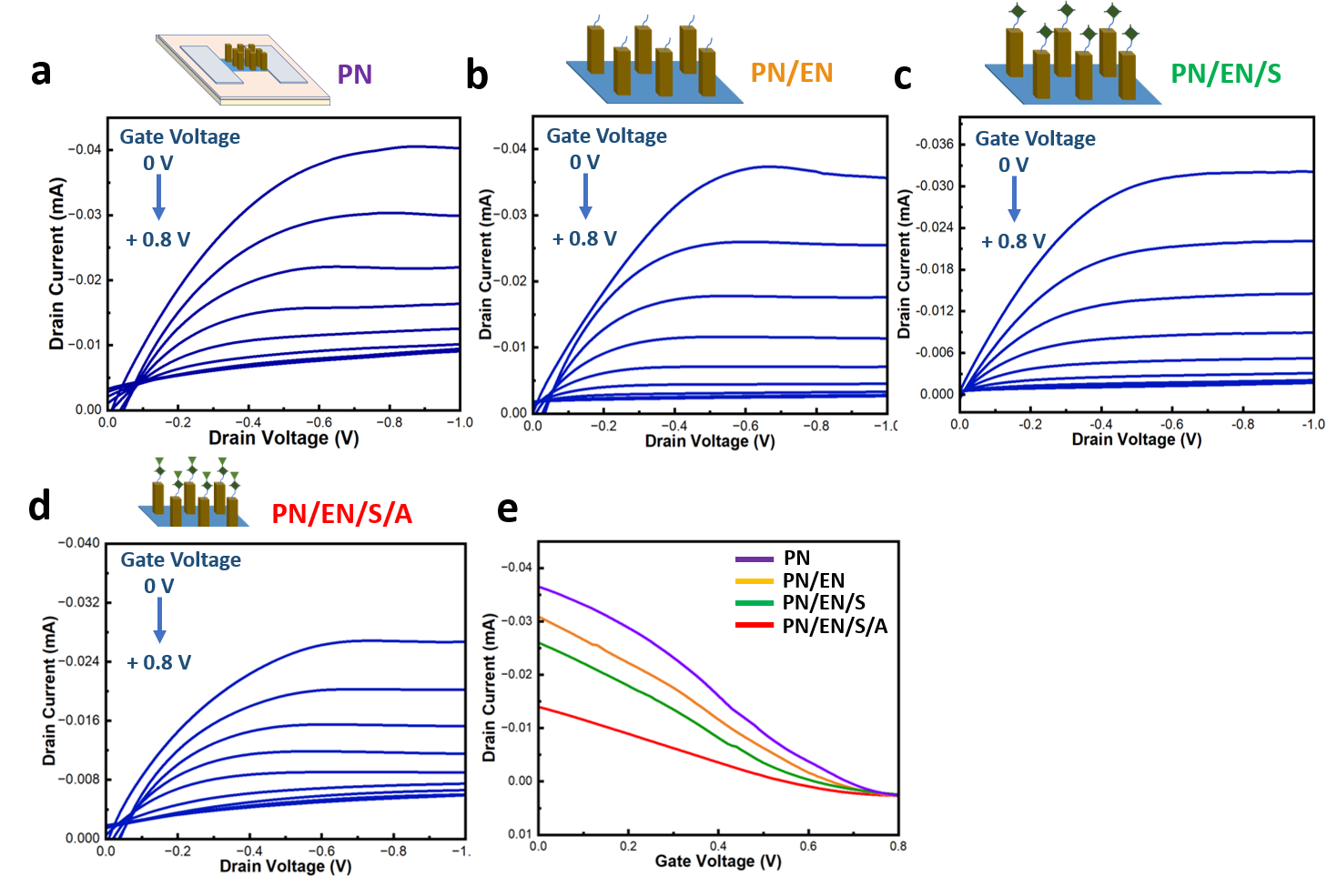


**Figure S5.** Electrical characteristics of PN-based OECT sensor. (a−d) Output characteristics of the OECT device (*I_d_–V_d_*) featuring PN fabricated using a template-based method after immobilizing with various linkers and then immobilizing with the receptor. (e) Drain current response concerning changes in gate voltage (*I_d_–V_g_*) of OECT device.


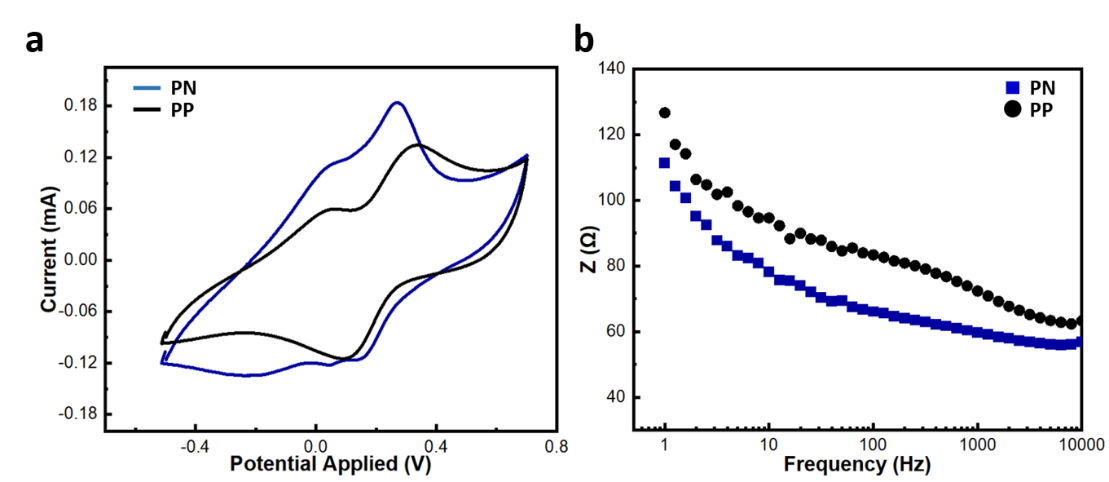


**Figure S6.** Electrical properties of PP and PN coated on patterned ITO glass in 0.1 M PBS. (a) CV potential swept from −0.8 to 0.8 V at a scan rate of 10 mV s^−1^. (b) Impedance responses of the surface of OECT channels with different linker modifications. The impedance responses were recorded at frequencies of 0.1 to 10000 Hz and 0.25 V.


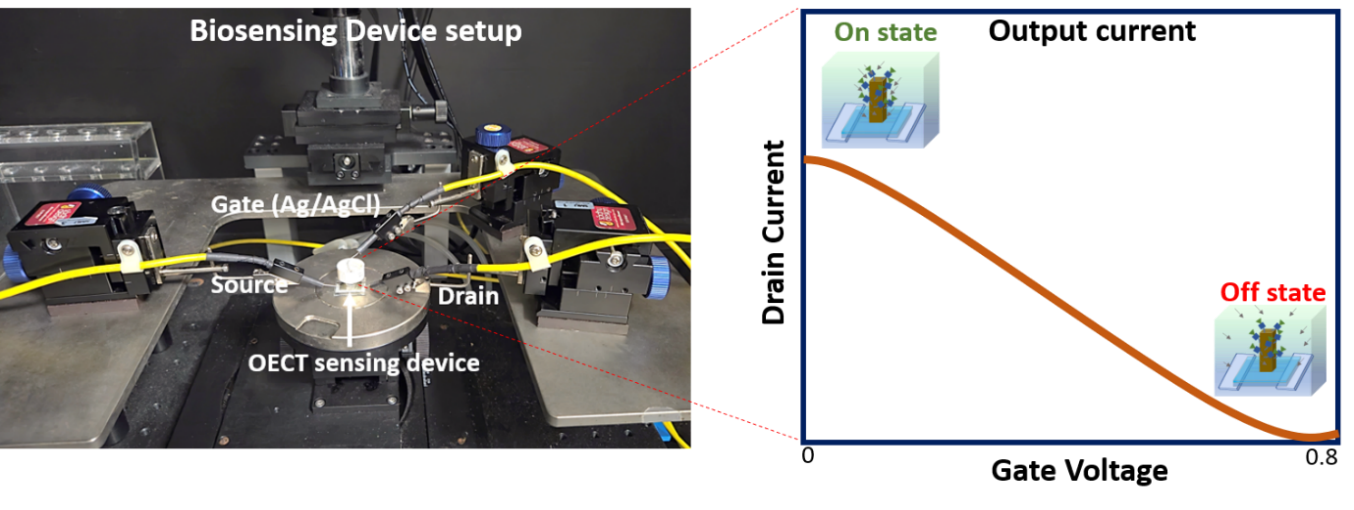


**Figure S7.** Device measurement for the current recording of an OECT-based sensor using Ag/AgCl as a gate electrode to detect target S1 protein. Schematic of the mechanism of the device in the on-off state as shown in the transfer curve.


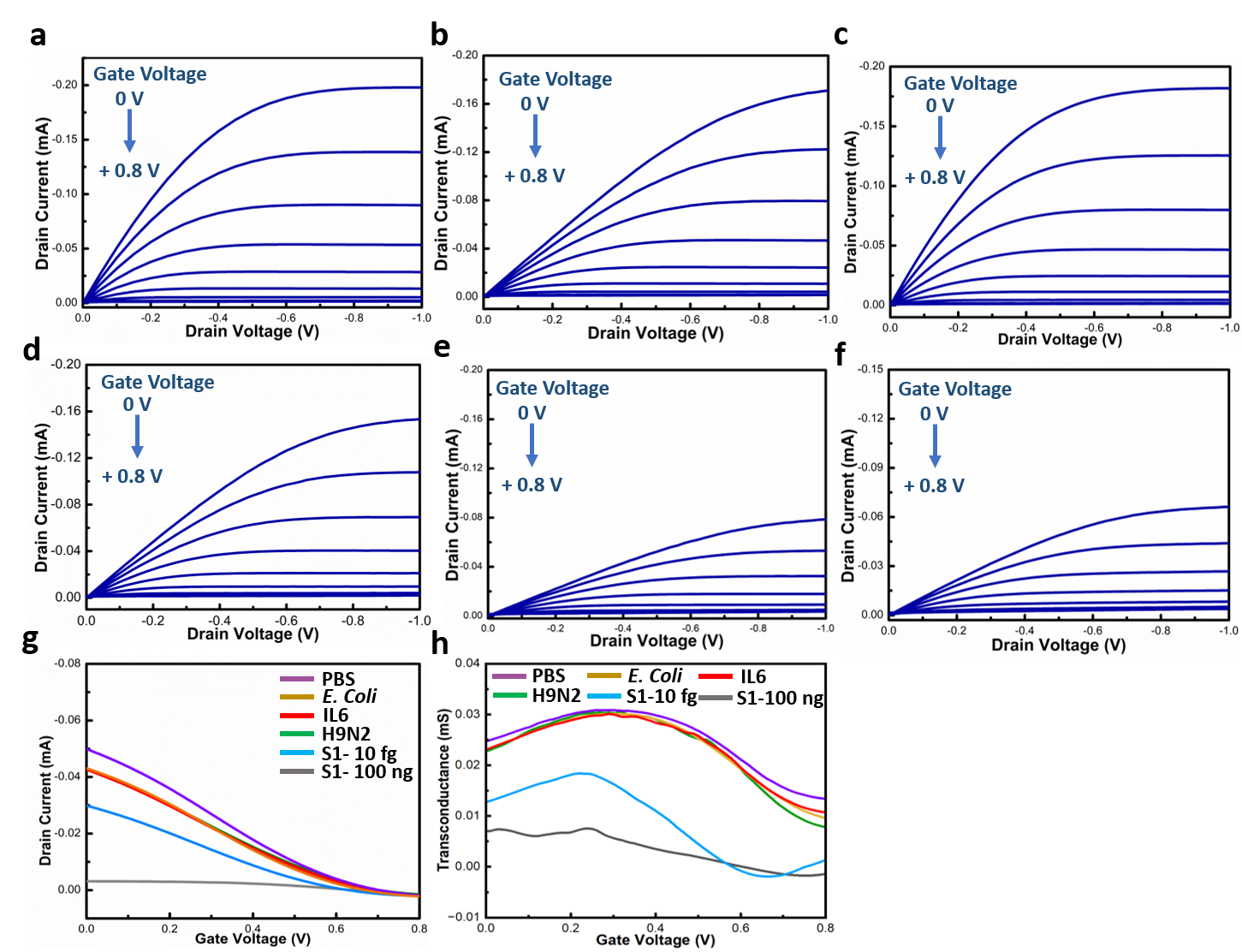


**Figure S8.** (a−f) Output curves of OECT devices show a specificity study with different non-specific proteins. (g, h) Transfer curves and transconductance of the OECT device for specificity study.


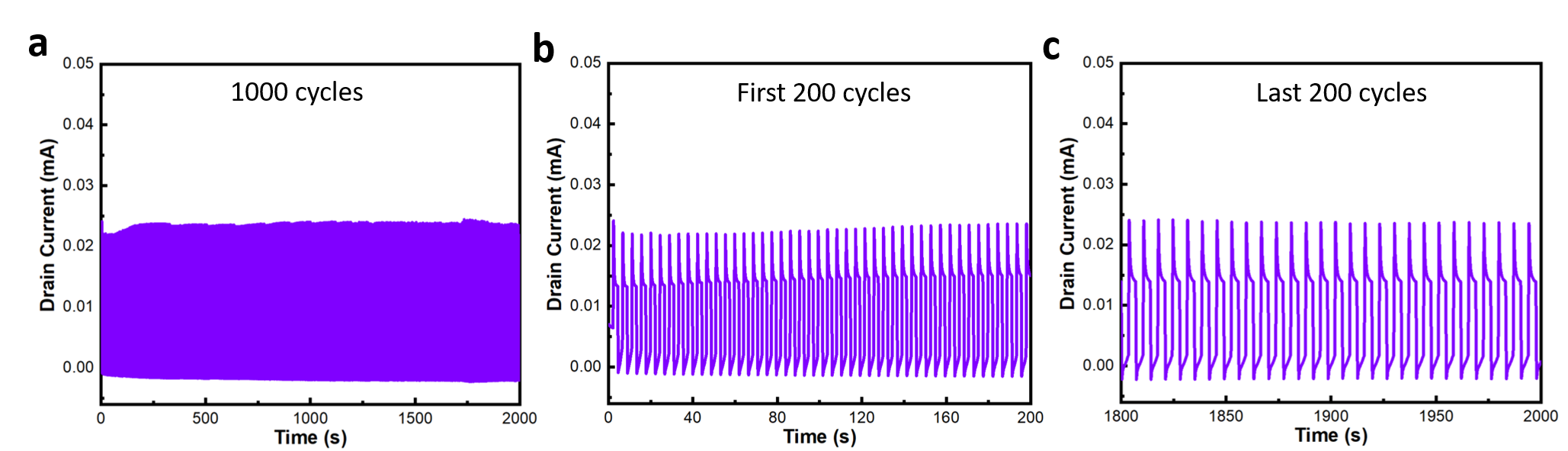


**Figure S9.** Long-term (*I_d_*−*Time*) stability was conducted on 3D nanorods-based OECTs in 1× PBS (pH 7.2) buffer solution. (a) Drain current-time response of the OECT device with applied gate voltage pulse 0.1 V (vs Ag/AgCl) with 1s pulse interval time (V_d_= -0.4 V) for the 2000s (1000 cycles) and (b) initial 200s time interval and (c) last 200 cycles result.

**Table S1.** Comparison to other previously reported protein sensors with the current study sensor based on the OECT device.

| Sensor Type | Detection Method | LOD | Ref. |
| --- | --- | --- | --- |
| hACE2-based peptide | Colorimetric | 0.01 nM | [S1] |
| Antibody-based AuNPs/ screen-printed gold electrode | Square wave voltammetry (SWV) | 1 pg/mL | [S2] |
| Fe_3_O_4_@SiO_2_–Au/GCE electrode | Impedance | 4.78 pg/mL | [S3] |
| Aptamer-based gold electrode | SWV | 50.4 pM | [S4] |
| CNT-Nanobodies | Field effect transistor (FET) | 1 pM | [S5] |
| CNT | FET | 7 fM | [S6] |
| Quantum Dots | Fluorescence resonance energy transfer (FRET) | 0.05 ng/mL | [S7] |
| PEDOT-NRs/AuNPs electrode | Differential pulse voltammetry (DPV) | 2 pg/mL | [S8] |
| PEDOT-NRs-based OECT channel | OECT | 0.1 fM | This work |

**References**

[S1] Hsiao, Y. S.; Luo, S. C.; Hou, S.; Zhu, B.; Sekine, J.; Kuo, C. W.; Chueh, D. Y.; Yu, H. H.; Tseng, H. R.; Chen, P. L. 3D Bioelectronic Interface: Capturing Circulating Tumor Cells onto Conducting Polymer-Based Micro/Nanorod Arrays with Chemical and Topographical Control. *Small* **2014**, *10*, 3012-3017.

1. Zhu, Q.; Zhou, X. H. A colorimetric sandwich-type bioassay for SARS-CoV-2 using a hACE2-based affinity peptide pair. *J. Hazard. Mater.* **2022**, *425*.
2. Karakus, E.; Erdemir, E.; Demirbilek, N.; Liv, L. Colorimetric and electrochemical detection of SARS-CoV-2 spike antigen with a gold nanoparticle-based biosensor. *Anal. Chim. Acta* **2021**, *1182*.
3. You, X. H. L., Y.; Li, Y. Y.; Zhao, B.; Yang, Y.; Weerasooriya, R.; Chen, X. Sensitive detection of SARS-CoV-2 spike protein based on electrochemical impedance spectroscopy of Fe3O4@SiO2–Au/GCE biosensor. *Adv. Sens. Energy Mater.* **2023**, *2*, 100067.
4. Idili, A.; Parolo, C.; Alvarez-Diduk, R.; Merkoçi, A. Rapid and Efficient Detection of the SARS-CoV-2 Spike Protein Using an Electrochemical Aptamer-Based Sensor. *ACS Sens.* **2021**, *6*, 3093-3101.
5. Filipiak, M. S.; Rother, M.; Andoy, N. M.; Knudsen, A. C.; Grimm, S.; Bachran, C.; Swee, L. K.; Zaumseil, J.; Tarasov, A. Highly sensitive, selective and label-free protein detection in physiological solutions using carbon nanotube transistors with nanobody receptors. *Sens. Actuator B-Chem.* **2018**, *255*, 1507-1516.
6. Kim, J. P.; Lee, B. Y.; Hong, S.; Sim, S. J. Ultrasensitive carbon nanotube-based biosensors using antibody-binding fragments. *Anal. Biochem.* **2008**, *381*, 193-198.
7. Li, Y.; Ren, Y. S.; Yi, Z. H.; Han, S. T.; Liu, S. L.; Long, F.; Zhu, A. N. Detection of SARS-CoV-2 S protein based on FRET between carbon quantum dots and gold nanoparticles. *Heliyon* **2023**, *9*.
8. Ali, S. A.; Ayalew, H.; Gautam, B.; Selvaraj, B.; She, J. W.; Janardhanan, J. A.; Yu, H. H. Detection of SARS-CoV-2 Spike Protein Using Micropatterned 3D Poly(3,4-Ethylenedioxythiophene) Nanorods Decorated with Gold Nanoparticles. *ACS Appl. Mater. Interfaces* **2024**, *16*, 19904–19913.
